# A New Paradigm for an Old Story: The Role of Regulatory B Cells in Cancer

**DOI:** 10.3389/fimmu.2012.00206

**Published:** 2012-07-23

**Authors:** Arya Biragyn, Catalina Lee-Chang

**Affiliations:** ^1^Immunotherapeutics Section, Laboratory of Molecular Biology and Immunology, National Institute on AgingBaltimore, MD, USA

A common feature between cancer escape and autoimmune diseases is an inappropriate involvement of the regulatory immune system, albeit for opposing purposes. While autoimmune disease is a reflection of the failure to control responses to self, cancer is a result of an exaggerated use of these controls to abrogate antitumor effector responses. Although the importance of regulatory B cells [Bregs, the definition first used by Mizoguchi to describe B cells exerting protection from colitis in mice (Mizoguchi et al., 1997)] in protection from autoimmunity is now accepted, their involvement in cancer escape remains poorly understood. The conundrum of Bregs is that, if their numbers are low (in analogy with Tregs), their existence and importance may be concealed by the overwhelming response of effector B cells. For example, aberrant activation of B cells promotes autoimmune diseases, such as rheumatoid arthritis (RA), type 1 diabetes mellitus (T1D), multiple sclerosis (MS), and systemic lupus erythematosus (SLE). As such, the depletion of B cells with anti-CD20 antibody rituximab impairs antigen-specific CD4^+^ T cell activation (Bouaziz et al., 2007) and ameliorates RA, MS, and T1D (Townsend et al., 2010). Yet, treatment with rituximab can also exacerbate the disease in some patients with ulcerative colitis, or even induce other diseases, such as psoriasis with psoriatic arthropathy and colitis in patients with Graves disease and non-Hodgkin lymphoma, respectively (Dass et al., 2007; Goetz et al., 2007; Mielke et al., 2008). The increased numbers of B cells in peripheral blood of transplant patients is positively associated with a rare but long-term drug-free clinical tolerance (Newell et al., 2010; Pallier et al., 2010; Sagoo et al., 2010). Although these clinical examples clearly indicate the importance of B cells, a current issue is how to segregate the role of Bregs from suppressive activity of B cells that has been known for more than 30 years. As first proposed by Morris and Moller in late 1960s (Morris and Moller, 1968), B cell-produced immunoglobulin can elicit immune suppression by directly triggering ITIM-mediated suppressive signaling in target cells upon binding with inhibitory FcγRIIB (Ravetch and Bolland, 2001) or by indirectly modulating dendritic cells (DCs) via activating FcγR (Morris and Moller, 1968).

The first evidence of suppressive B cells (Bregs?) that functioned independently of their immunoglobulin was shown by Shimamura et al. (1982) about 30 years ago. Confirming this, the absence of B cells was linked with exacerbated autoimmune responses in mice deficient in B cells, such as mice that lack mature B cells (Wolf et al., 1996) and CD19 B cells (Yanaba et al., 2008). To date, the protection from autoimmune diseases in mice was linked with several unique subsets of IL-10-producing Bregs, such as CD1d^High^ B1b cells (CD5^−^ B220^Low^ CD11b^+^ IgM^+^ CD1d^High^; Mizoguchi et al., 2002), B10 regulatory cells (IL-10-producing CD1d^High^ CD5^+^ B cells; Yanaba et al., 2008), and CD1d^High^ Tim-1^+^ CD5^+^ Bregs (Ding et al., 2011). Although little is known about human Bregs, protection from SLE was recently linked with an impairment of regulatory activity of CD19^+^ CD24^High^ CD38^High^ B cells (Blair et al., 2010). Moreover, a rare subset of IL-10-producing memory CD24^hi^ CD27^+^ B cells that functions like murine B10 cells was also shown to exist in humans (Iwata et al., 2011). Humans also have IL-10 and TGFβ-producing CD25^hi^ CD27^hi^ CD86^hi^ CD1d^hi^ B cells that can suppress proliferation of autologous T cells and induce the generation of Foxp3^+^ CTLA-4^+^ Tregs (Kessel et al., 2012).

The majority of protective effects of Bregs requires IL-10 (Mizoguchi et al., 2002; Byrne and Halliday, 2005; Matsushita et al., 2008; Yanaba et al., 2008; Blair et al., 2010), a cytokine also utilized in other B cell-mediated suppression. For example, IL-10 is also abundantly produced and utilized by CD5^+^ B1 cells and MZ B cells to ameliorate collagen-induced arthritis in mice (O'Garra and Howard, 1992; Brummel and Lenert, 2005; Lenert et al., 2005; Evans et al., 2007) and by LPS-stimulated B cells to protect from autoimmune responses in mice by rendering T cells anergic (Parekh et al., 2003; Lampropoulou et al., 2008) and tolerogenic (Fuchs and Matzinger, 1992). The boundaries between Bregs and IL-10 producing B cells can often be obscure, raising question whether IL-10 is a primary mediator of suppressive activity or a factor that promotes homeostasis of Bregs. As for murine and human B1 cells (Balabanian et al., 2002; Gary-Gouy et al., 2002), IL-10 may promote survival and proliferation of Bregs. On the other hand, full suppressive power of Bregs and concomitant IL-10 production often requires activation, for example, by chronic inflammation or by engagement of their toll-like receptors (TLRs) or CD40 (Mizoguchi et al., 2002; Gray et al., 2007; Lampropoulou et al., 2008). This leads to production of other immunomodulatory factors (TGFβ and galectin-1) and upregulation of surface antigens, such as PD-1 and CTLA-4. As a result, activated Bregs can either directly induce apoptosis and anergy of effector Th1 cells and CD8^+^ T cells (Zuniga et al., 2001; Parekh et al., 2003; Frommer et al., 2008; Tretter et al., 2008) or indirectly by converting Tregs (Reichardt et al., 2007; Sun et al., 2008; Sayi et al., 2011; Scapini et al., 2011) and modulating DCs (Byrne and Halliday, 2005; Watt et al., 2007).

Although cancer often uses homeostatic regulatory machinery to escape from immune surveillance, surprisingly the process seems does not involve Bregs that protect from autoimmune diseases. As such, the role of Bregs in cancer escape is poorly appreciated. Instead, B cells are mostly known for their “pathogenic” antitumor properties (Lanzavecchia, 1985; Candolfi et al., 2011). The presence of CD20^+^ B cells in metastatic lymph nodes is a sign of favorable outcome in patients with head and neck cancer (Pretscher et al., 2009); and depletion of CD20-expressing B cells increases tumor burden in the lungs of mice intravenously injected with B16-F10 melanoma after (Sorrentino et al., 2011). Despite this, B cells also participate in carcinogenesis of methylcholanthrene-induced (Brodt and Gordon, 1978, 1982) or transplanted tumors (Monach et al., 1993); and syngeneic tumors progress poorly in μMT mice deficient in B cells unless replenished with B220^+^ B cells (Qin et al., 1998; Olkhanud et al., 2011). Cancer-promoting B cells appear to exert a multitude of functions, such as production of immunoglobulins and cytokines (Townsend et al., 2010). As in autoimmunity, the immunoglobulin deposition induces FcR- and complement-mediated chronic inflammation needed for carcinogenesis (Zusman et al., 1996; de Visser et al., 2005). Activated B cells produce TGFβ (Parekh et al., 2003; Lampropoulou et al., 2008), and immunoglobulin can serve as a carrier for TGFβ and thereby mediate suppression of cellular immune responses (Stach and Rowley, 1993; Rowley and Stach, 1998). Tumor-infiltrating B cells also produce lymphotoxin α/β and promote androgen-independent growth of prostate cancer cells by inducing the nuclear translocation of IKKα and activation of STAT3 (Ammirante et al., 2010). Pro-tumorigenic activity of B cells also requires production of IL-10 (Inoue et al., 2006) and TNFα (Schioppa et al., 2011) to presumably mediate Th2 polarization and inhibition of the cytotoxic activity of CD8^+^ T and NK cells. Importantly, B cells isolated from tumor-bearing mice inhibit CD4^+^ T cell-mediated help for CTLs (Qin et al., 1998).

To date, there are only two clearly defined examples of cancer escape-promoting Bregs are reported. First, murine B10 cells can abrogate monocyte activity and reduce surface expression of FcγR in IL-10-dependent fashion (Horikawa et al., 2011). As a result, the presence of B10 cells inhibits the therapeutic efficacy of anti-CD20 antibody against lymphoma. On the other hand, we recently discovered a unique subset of tumor-evoked Bregs (tBregs) that actively facilitates breast cancer escape and metastasis in BALB/C mice bearing 4T1 carcinoma cells (Olkhanud et al., 2011). In fact, the cancer cells themselves induce the generation of TGFβ-producing tBregs from normal B cells. As a result, tBreg then convert non-Treg CD4^+^ T cells into metastasis-promoting FoxP3^+^ Tregs (Olkhanud et al., 2011), which in turn inactivate antitumor NK cells and protect metastasizing cancer cells in the lungs (Olkhanud et al., 2009). We believe that the tBreg-like cells also exist in humans, as they can be readily generated *ex vivo* by treating normal human donor B cells with conditioned media of human cancer lines, such as breast, ovarian, and colon carcinomas (Olkhanud et al., 2011). tBregs differ phenotypically and functionally from other Bregs involved in autoimmune responses (Mizoguchi et al., 2002; Matsushita et al., 2008; Yanaba et al., 2008) and LPS- or BCR-activated B cells (Fuchs and Matzinger, 1992; Hussain and Delovitch, 2007). tBregs resemble B2 cells (IgD^High^) but express constitutively active Stat3 and surface markers like CD25^High^ B7-H1^High^ CD81^High^ CD86^High^ CCR6^High^ and CD62L^Low^ IgM^Int/Low^ and poorly proliferate. They do not express CD27 and CD5 or up regulate CD1d, and their suppressive activity does not require IL-10 or other known suppressive pathways, such as B7-H1-PD-1, Fas-FasL, and IL27/IL35. Treatment with *S. aureus* Cowan 1 antigen can also generate suppressive CD25^+^ B cells that induce anergy of activated T cells by competing for IL-2 (Tretter et al., 2008). However, unlike them, tBregs regulate both resting and activated T cells (both CD4^+^ and CD8^+^ T cells) acting independently of IL-2 and without inducing cell death.

Since cancer actively converts tBregs from normal B cells, the clinical implication of this is that, as long as cancer persists, it will induce their generation and thereby initiate the chain of suppressive events. Thus, strategies that abrogate any step of this process are expected to inhibit cancer escape and metastasis, a primary cause of patients’ bad disease outcome. However, the success of a strategy will also depend on the use of tailored approaches, ideally, ones that only inactivate tBregs, while protecting or promoting “good” B cells needed for optimal cancer eradication. For example, 4T1 breast cancer metastasis is abrogated by antibody that targets IL2Rα expressed on Tregs and tBregs (Olkhanud et al., 2009, 2011). Despite this, no clinical benefit was elicited in patients with renal cell carcinoma treated with B cell-depleting anti-CD20 antibody rituximab (Aklilu et al., 2004). Although this result questions the role of Bregs in human cancers, our recent data indicate that tBregs can escape anti-CD20 antibody due to low levels of CD20 expression. As a result, treatment with anti-CD20 antibody preferentially depletes “good” and activated B cells, while enriching for tBregs and thereby enhancing cancer escape and metastasis (Bodogai et al., MS in preparation). Overall, although plethora of conventional B cells can often conceal and hamper analysis of small population of Bregs, the use of tailored and unique methodologies clearly indicates their existence and importance in mediation of cancer escape. It is time to unequivocally accept Bregs and tBregs as true members of the regulatory immune network.
